# Prevalence of extended-spectrum beta-lactamase and molecular detection of blaTEM, blaSHV, and blaCTX-M genotypes among gram-negative Bacilli isolates from hospital acquired infections in pediatrics, one institutional study

**DOI:** 10.1186/s13052-024-01599-9

**Published:** 2024-02-24

**Authors:** Ahmed Gomaa Ahmed Elsayed, Dina F. Badr, Nermene Youssef Abo El Kheir, Maysaa El Sayed Zaki, Abdelrahman Eid Mahmoud Mossad, Ehab Mohammed Fahmy Mahmoud

**Affiliations:** 1 Medical Microbiology and Immunology, Mansoura Faculty of Medicine, Mansoura, Egypt; 2 Clinical Pathology Department, Mansoura Faculty of Medicine, Mansoura, Egypt; 3 Pediatrics Department, Mansoura Faculty of Medicine, Mansoura, Egypt; 4 Medical Microbiology and Immunology Department, Helwan Faculty of Medicine, Helwan, Egypt

**Keywords:** ESBL, Phenotypic, Multiplex PCR, BlaCTX-M, BlaSHV, BlaTEM

## Abstract

**Background:**

Gram-negative bacilli represents an important pathogen in hospital-acquired infections (HAIs) worldwide. The emergence of antibiotic resistance in these pathogens warrants attention for the proper management of infections. Extended-spectrum beta-lactamase (ESBL) resistance represents a major therapeutic problem in infections due to Gram-negative bacilli.

The present study aimed to study the extended-spectrum beta-lactamase genes blaTEM, blaSHV, and blaCTX-M by multiplex polymerase reaction in isolated Gram-negative bacilli from HAIs in pediatric patients.

**Methods:**

The study included one hundred-five isolates of Gram-negative bacilli from pediatric patients with different types of HAIs. The isolates were subjected to full microbiological identification, antibiotics susceptibility by disc diffusion method, the phenotypic study of ESBL, and the genetic study of ESBL genes by multiplex PCR.

**Results:**

Fifty isolates of Gram-Negative bacilli showed ESBL activity by a phenotypic study by double disc diffusion method (50/105). All ESBL producers’ isolates were positive by PCR for ESBL genes. The most frequent gene was blaTEM (64%), followed by blaSHV (30%) and CTX-M (22%). Mixed genes were found in 4 isolates (8%) for blaTEM and blaSHV, blaTEM and CTX-M. There was a significant association between PCR for ESBL genes and phenotypic ESBL detection (*P* = 0.001). There was significant detection of ESBL genes in *E. coli* (28%), followed by *Enterobacter* spp. (26%), *Klebsiella* spp. (24%), *Serratia* (14%), *Pseudomonas* spp. (6%) and *Proteus* (2%), *P* = 0.01. There Seventy percent of isolates positive for ESBL production had an insignificant association between MDR and PCR for ESBL genes (*P* = 0.23).

**Conclusion:**

The present study highlights the prevalence of ESBL activity among clinical isolates of Gram-negative bacilli isolated from hospital-acquired infections in pediatric patients. The most common gene responsible for this activity was blaTEM gee followed by blaSHV and blaCTX-M. There was a high prevalence of multiple antibiotic resistance among isolates with ESBL activity. The finding of the present study denotes the importance of screening extended beta-lactamase among Gram-negative bacilli associated with HAIs in pediatric patients.

## Introduction

Hospital-acquired infection is a global health problem with around two and a half million new infections every year in European countries with a fourth of them being due to multidrug-resistant microorganisms (MDR) [1, 2]. Gram-negative bacilli is an important etiology of such infections with species of Enterobacteriaceae family such as *E.coli* and *Klebsiella* species representing principle etiology in many geographical regions such as United States of America, Canada, Middle East, Europe, Asia, and Australia [3] Pediatric patients are susceptible to HAIs due to their immature immune systems, presence of multiple comorbidities, and cross-infection from recurrent close contact with health care team members [4].

Gram-negative bacilli had shown resistance to antibiotics with broad-spectrum activity due to extended-spectrum β-lactamases (ESBLs) those results from the acquiring the gene coding enzymes that increase the efflux pumps leading to the changes in the antibiotic binding sites [5]. The antibiotic of choice to treat these infections is carbapenems [6].

There are many types of ESBL genes with *TEM* and *SHV* as the most prevalent resistance genes however, there is a reported increase in the CTX-M gene [7–9]. The CTX-M β-lactamase gene was reported in 1980 with more than 100 variants reported [10].

The prevalence of the ESBL genotypes varied by geographical region and the time of infection. The predominant types of ESBL genes were SHV and TEM in the United States of America and Europe during the period from 1980 up to 1990 [11, 12], while in Asia the most prevalent ESBL genotype was CTX-M [13, 14]. The last two decades have shown an increase in the CTX-M genotype variants CTX-M15 and CTX-M14 [15]. The epidemiology of ESBL genotyping in adult patients is well studied in various reports [16–19] and Egypt [20]. However, fewer studies reported ESBLs in pediatric patients.

Therefore, the present study aimed to study the ESBL genes TEM, SHV, and CTX-M by multiplex polymerase reaction in isolated Gram-negative bacilli from HAIs in pediatric patients.

## Material and method

The study was a retrograde cross-sectional study that included one hundred- and five-gram negative isolates from pediatric patients with hospital-acquired infections from January 2019 till January 2020. The children were diagnosed with HAIs as described by criteria of the Centers for Disease Control (CDC) guidelines [21]. Children with community-acquired infections and children with HAIs associated with Gram-positive isolates were the exclusion criteria for this study. The ethical approval of the study was obtained from the ethical committee of Mansoura Faculty of Medicine (R.23.09.2330) and written approval was obtained from the parent of each child.

### Bacterial identification

After primary isolation of organisms from the clinical samples, further identification was performed according to standard microbiological methods. Each isolate was cultured onto Trypticase soy agar with 10% sheep blood with incubated at 35 °C in an atmosphere with 5% CO_2_ for 72 hours. Then identification was performed by Gram stain and an oxidase test, followed by Vitek 2 (bioMérieux-USA) automated identification systems to achieve a species-level identification [22].

### Antibiotics sensitivity test by disc diffusion method

The used antibiotics discs were imipenem (10 μg), cefepime (30 μg), amikacin (30 μg), amoxicillin/clavulinic acid (20/10 μg), ampicillin (10 μg), aztreonam (30 μg), cefotaxime (30 μg), cefoxitin (30 μg), ceftazidime (30 μg), ceftriaxone (30 μg) gentamicin (10 μg), piperacillin (100 μg), piperacillin/tazobactam (100/10 μg), cefoperazone (75 μg), ciprofloxacin (5 μg), gatifloxin (5 μg), amoxicillin (5 μg) (Oxoid, United Kingdom). Gram-negative bacilli isolates were suspended in Muller-Hinton broth for preparation of 0.5 McFarland concentrations then spread over Muller-Hinton agar. The discs were applied over the agar then the plates were incubated at 37 °C for 24 hours. The measured inhibition zone diameter around the discs was interpreted as sensitive or resistant according to the guidelines of the Clinical Laboratory Standards Institute (CLSI) [23]. Multidrug resistance (MDR) was identified as resistance to three or more of antibiotic classes.

### Detection of ESBL

Gram-negative bacilli isolate resistant to ceftazidime or/or cefotaxime were further tested for the ESBL phenotype by double discs method [23]. The isolates were diluted in Muller-Hinton broth to prepare o.5 McFarland and plated over a Muller-Hinton agar plate and cefotaxime and ceftazidime discs and ceftazidime compound with clavulanic acid discs were added with incubation at 37 °C for 24 hours. The interpretation of ESBL was reported if there was an increase of the zone of inhibition around combined antibiotics discs with clavulanic acid by ≥5 mm. The used organism as negative control for ESBL was *Klebsiella pneumoniae* ATCC 700603 and the used organism as positive control was *E. coli* ATCC 25922.

### Multiplex PCR for detection of TEM, SHV, and CTX-M genes

#### DNA extraction method

DNA was extracted from isolated pure colonies by heat method [24]. Colonies were obtained from culture over a MacConkey plate and suspended in 40 μm of sterile distilled water. The suspension was incubated at 95 °C for 5 minutes and then centrifuged at 12000 rpm for 10 minutes. Then the supernatant was obtained and stored at − 20 °C till the time of amplification.

#### Multiplex PCR

Five microns of the extracted DNA was emulsified in the 50 μl reaction mix, containing 10 pmol of the used primers, Table 1, 10 mM dNTPs mix, and 2.5 U of Taq polymerase (Qiagen, Hilden, Germany) in 1x Taq polymerase buffer. The used negative control was *E. coli* ATCC 25922. The sequences of the amplifications were heating at 94 °C for 5 minutes for denaturation, then 35 cycles including heating at 94 °C for 30 seconds, followed by heating at 60 °C for 30 seconds heating at 72 °C for 50 seconds, and finally extension for one cycle at 72 °C for 5 minutes. The PCR product was electrophoresed using 1.5% agarose gel with ethidium bromide to visualize the amplified fragment [25].

**Table 1:** Genes and the sequences of the primers and base pair(bp)

Gene	Sequences of the primers	Bp
TEM	TTTCGTGTCGCCCTTATTCC404 ATCGTTGTCAGAAGTAAGTTGG	404
SHV	CGCCTGTGTATTATCTCCCT CGAGTAGTCCACCAGATCCT	294
CTX-M	CGCTGTTGTTAGGAAGTGTG GGCTGGGTGAAGTAAGTGAC	754

### Statistical analysis

The data was analyzed using the SPPS 22.0 package. Quantitative data was interpreted as mean and standard deviation (SD). Qualitative data was interpreted as number and percentage and the comparison was performed by Chi-square test. *P* was considered significant if < 0.05.

## Result

The study included 105 pediatric patients with an age range of 0.2 up to 15.5 years. They were 48.6% males and 51.4% females. The most frequent infections were urinary tract infections 50.5%, followed by respiratory tract infections (22.9%) wound infections (15.2% and sepsis (11.4%), Table 2.

**Table 2:** Demographic and clinical data of the studied pediatric patients

Age (years)		
Minimum	0.2 years	
Maximum	15.5	
Median	6.00	
Sex		
Male (No.-%)	51	48.6%
Female (No.-%)	54	51.4%
Urinary tract infections (No.-%)	53	50.5%
Respiratory tract infections (No.-%)	24	22.9%
Wound Infections (No.-%)	16	15.2%
Sepsis (No.-%)	12	11.4%

**Fig. 1:**
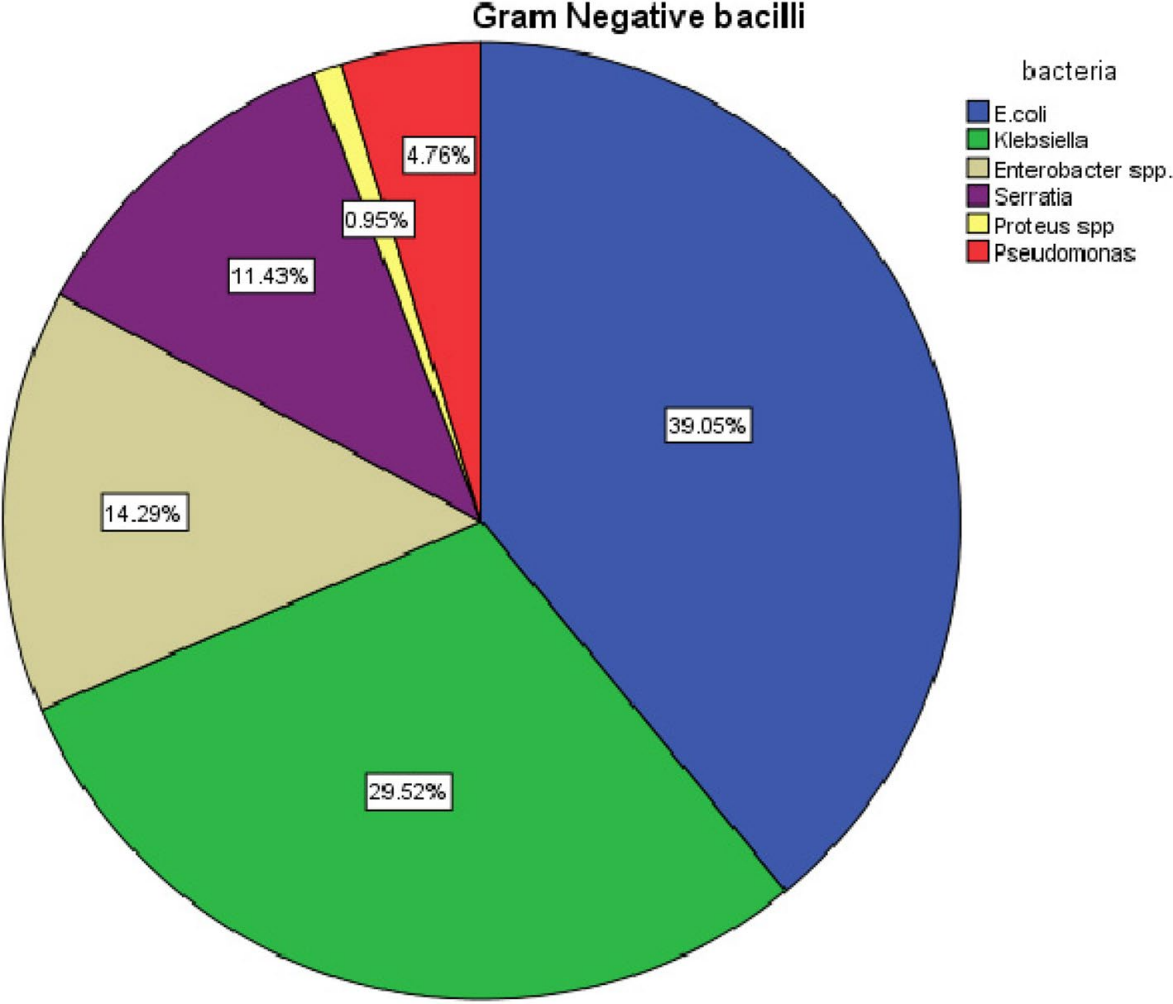
Frequency of the isolated Gram-Negative bacilli

The most prevalent Gram-negative bacilli isolated was *E. coli* (39%), followed by *Klebsiella* spp. (29.5%0, *Enterobacter* spp. (14.3%), and *Serratia (*11.4%). *Pseudomonas spp* was isolated from 4.8% of the samples, Fig. 1.

The most antibiotic resistance of the isolated Gram-negative bacilli was for beta-lactam antibiotics, amoxicillin (94.3%), ampicillin (81.9%), ampicillin (81.9%), cefoperazone (81.0%), Amoxicillin/clavulanic acid (73.3%), piperacillin (67.6%). The least antibiotic resistance was for amikacin (12.4%), gatifloxacin (33.3%), and garamicin (36.2%). Resistance to imipenem was 44.8%, Table 3.

**Table 3:** Antibiotic resistance of isolated Gram-Negative bacilli

Antibiotics	No.	%
Amoxicillin	99	94.3
Ampicillin	86	81.9
Aztreonam	58	55.2
Cefotaxime	68	64.8
Ceftriaxone	62	59.0
Ceftazidime	69	65.7
Cefoperazone	85	81.0
Imipenem	47	44.8
Piperacillin	71	67.6
Amoxicillin/clavulanic acid	77	73.3
Amikacin	13	12.4
Garamicin	38	36.2
Ciprofloxacin	46	43.8
Gatfloxacin	35	33.3
Cefoxitin	61	58.1
MDR	67	63.8

Fifty isolates of Gram-Negative bacilli showed ESBL activity by a phenotypic study by double-disc diffusion method (50/105). All ESBL producers’ isolates were positive by PCR for ESBL genes. The most frequent gene was blaTEM (64%), followed by blaSHV (30%) and CTX-M (22%). Mixed genes were found in 4 isolates (8%) for each of blaTEM and blaSHV, and CTX-M and blaTEM. There was a significant association between PCR for ESBL genes and phenotypic ESBL detection (*P* = 0.001), Table 4.

**Table 4:** Association between phenotypic ESBL detection and multiplex PCR for ESBL genes

Multiplex-PCR	ESBL			
	Positive (no = 50)		Negative (*n* = 55)	
	N0.	%	N0.	%
Multiplex-PCR	50	100	55	100%
blaTEM	32	64	0	0%
blaSHV	15	30	0	0%
CTX-M	11	22%	0	0%
Mixed TEM and SHV	4	8%		
Mixed CTXM and TEM	4	8%		
Mixed SHV and CTX-M	0	0%		

Chi-square, *P* = 0.001.

There was significant detection of ESBL genes in *E. coli* (28%), followed by *Enterobacter* spp. (26%), *Klebsiella* spp. (24%), *Serratia* spp. (14%), *Pseudomonas* spp. (6%) and *Proteus* spp. (2%), *P* = 0.01, Table 5.

**Table 5:** ESBL genes in isolated bacteria

Organisms	PCR positive for ESBL genes (*n* = 50)	
	No.	%
*E. coli*	14	28
*Klebsiella* spp.	12	24
*Enterobacter* spp.	13	26
*Serratia*	7	14
*Pseudomonas* spp.	3	6
*Proteus* spp.	1	2

Chi-square test, *P* = 0.01.

There Seventy percent of isolates positive for ESBL production had an insignificant association between MDR and PCR for ESBL genes (*P* = 0.23), Table 6.

**Table 6:** Association between MDR and ESBL genes

MDR	PCR for ESBL genes			
	Positive (*n* = 50)		Negative (*n* = 55)	
	No.	%	No.	%
MDR				
Positive	35	70%	32	58.2%
Negative	15	30%	23	41.8%

Chi-square test, *P* = 0.23.

There was a statistically significant association between the presence of ESBL and stay in hospital for more than 7 days and mortality of the patients (*P* = 0.001), Table 7.

**Table 7:** Association between ESBL and outcome of the patients

		outcome					
		Death		less than 7 days		more than 7	
		No.	%	No.	%	No.	%
ESBL	positive	8	100	2	3.5	40	100
	negative	0	0	55	96.5	0	0
Total		8	100	57	100	40	100

Chi-square test, *P* = 0.001.

## Discussion

Pediatric patients are vulnerable to hospital-acquired infections attributed to their immature system, the presence of underlying comorbidities, and close contact with healthcare personnel [4].

During the period of the present study, the most frequent infections were urinary tract infections 50.5%, followed by respiratory tract infections (22.9%) wound infections (15.2%), and sepsis (11.4%). This finding was contrary to previous findings reporting that the most prevalent HAIs in pediatric patients were central line–associated bloodstream infection that ranges from 25% up to 30%, ventilator-associated pneumonia (VAP) with ranges from 20% up to 25%, urinary tract infection associated with catheter 15% [26], and surgical site infection (SSI) 11% [27]. Another study revealed The most common HAI was surgical site infection (40.0%), followed by bloodstream infection (21.5%), and lower respiratory tract infection (14.6%) [28]. The type of HAI differs according to the age of the patients, geographical regions, the practice of preventive measures, and the time of surveillance. The preventive measures for HAIs include the need for adequate isolation measures, proper sterilization of the devices, regular microbiologic audits, appropriate hand hygiene practices, and efficient education and training [29].

In the present study, the most prevalent Gram-negative bacilli isolated was *E. coli* (39%), followed by *Klebsiella* spp. (29.5%), *Enterobacter* spp. (14.3%), and *Serratia (*11.4%). *Pseudomonas* spp. Among the previous 19 studies from Egypt reporting the prevalence of Gram-negative bacilli infections among pediatric patients, *Klebsiella* species/*K. pneumoniae* and *Escherichia coli* were typically the most frequently isolated organisms Also, in Saudi Arabia, *Klebsiella* spp./*K. pneumoniae* and *E. coli* were the most frequently associated with infections [30].

The most antibiotic resistance of the isolated Gram-negative bacilli was for beta-lactam antibiotics, amoxicillin, ampicillin, ampicillin, cefoperazone, Amoxicillin/clavulanic acid, and piperacillin. The last antibiotic resistances were for amikacin (12.4%), gatifloxacin (33.3%), and garamicin (36.2%). Resistance to imipenem was 44.8% Previous report supported our finding as the resistance was high for multiple classes of beta-lactams (Antimicrobial Resistance Collaborators) and lower resistance of *E. coli* and *Klebsiella* spp. to amikacin and carbapenem antibiotics [31].

This finding denotes the importance of identifying methods that may reduce antimicrobial resistance in HAIs through the implementation of an antibiotics stewardship program and adequate antibiotics surveillance [32].

The ESBLs are identified as the ability of the organisms to hydrolyze various types of *β*-lactam antibiotics, including the third generation of cephalosporins such as cefotaxime, ceftriaxone, ceftazidime, and monobactams such as aztreonam. Gram-negative bacteria with ESBL capacity have significant therapeutic difficulties.

In the present study, around half of the isolates (50/105) had ESBL activity. In a previous study from Gaza, (51.6%) of Gram-negative bacilli were ESBL producers [33]. Significant prevalence of extended-spectrum beta-lactamase production from isolated Gram-negative bacilli was also reported in different Asian countries with varying ranges according to countries, reported to be 66.7% in India [34], 54.7–61% in Turkey [35, 36], 41% in United Arab Emirates [37], and 72.1% in Iran [38].

In the genetic study of ESBL genes, the most prevalent gene was blaTEM (64%), followed by blaSHV (30%) and blaCTX-M (22%). Mixed genes were found in 4 isolates (8%) for blaTEM and blaSHV, blaTEM and blaCTX-M. In the Previous report detection of these genes by PCR was 85 (59%) had at least one gene with the prevalence rates of blaCTX-M was 60%, blaTEM was 57.6%, and blaSHV was 38.3% [33].

Like our results, the previous report found that the blaTEM gene was the most prevalent (49%) followed by blaSHV (44%) and blaCTX-M (28%), [39]. On the other hand, previous studies revealed that the most prevalent genotypes of ESBL were blaTEM (86%), blaCTX-M (78%), and blaSHV (28%) [40]. The importance of genetic studies of ESBL genes is attributed to the capacity of these genes to spread horizontally to other bacterial species leading to widespread of ESBL activity in the hospital among different pathogens [40].

Seventy percent of isolates positive for ESBL genes detection by PCR were MDR, though this association was statistically insignificant, it is an alarming sign. The trend of multidrug-resistant profile associated with the currently analyzed genes *bla*TEM, *bla*HSV, and *bla*CTX-M to set up a routine screening of ESBL in clinical laboratories to prevent the spread of resistant isolates in health care settings. Previous data supported the association of ESBL and MDR among Gram-negative bacilli as a previous study reported that 53.3% of MDR *E. coli* were found resistant to > 7 antimicrobial agents and ESBL was detected in 32.7% of them [40, 41].

Clinical Isolates with ESBL activity are responsible for outbreaks in healthcare settings and lead to treatment failure with an increase in hospital cost and increased mortality rate due to treatment failure [42, 43]. In the present study, there was a statistically significant association with increased hospital stay and mortality rate with ESBL activity. The treatment of ESBL-producing isolates depends mainly on the use of Carbapenems. However, the resistance to carbapenem is increasing [44]. Therefore, the introduction of antimicrobial stewardship programs in healthcare is essential to overcome the growing rates of antimicrobial resistance [45].

## Conclusion

The present study highlights the prevalence of ESBL activity among clinical isolates of Gram-negative.

Bacilli isolated from hospital-acquired infections in pediatric patients. The most common gene responsible for this activity was blaTEM gee followed by blaSHV and blaCTX-M. There was a high prevalence of multiple antibiotic resistance among isolates with ESBL activity. The finding of the present study denotes the importance of screening extended beta-lactamase among Gram-negative bacilli associated with HAIs in pediatric patients.

## Data Availability

The datasets generated and analyzed during the current study are available in the Fighshare repository at 10.6084/m9.figshare.24153192.v1

## Abbreviations

ESBL: Extended spectrum beta lactamase
HAIs: Hospital-acquired infections
MDR: Multidrug-resistant microorganisms
